# Diagnostic accuracy of blood centers in the screening of blood donors for viral markers

**DOI:** 10.11604/pamj.2015.20.119.5263

**Published:** 2015-02-10

**Authors:** Elliot Eli Dogbe, Fareed Arthur

**Affiliations:** 1 Transfusion Medicine Unit, Komfo Anokye Teaching Hospital, Kumasi, Ghana; 2 Department of Biochemistry and Biotechnology, College of Science, Kwame Nkrumah University of Science and Technology, Kumasi, Ghana

**Keywords:** Transfusion transmitted infections, human immunodeficiency virus, hepatitis B virus, hepatitis C virus, sensitivity, specificity, Ghana

## Abstract

**Introduction:**

Blood transfusion still remains a life saving intervention in almost all healthcare facilities worldwide. Screening of blood donors/blood units is done in almost every blood bank facility before the blood units/blood components are transfused to prevent transfusion-transmissible infections. The kind of testing kits or the methods used by a facility and the technical expertise of the personnel greatly affects the screening results of a facility. This study was aimed at evaluating the diagnostic accuracy of five hospital-based blood bank testing facilities (Komfo Anokye Teaching Hospital KNUST, Kwame Nkrumah University of Science and Technology, Agogo, Bekwai and Sunyani) that used rapid immunochromatograhic assays (RIA) in screening blood donors/blood units in Ghana.

**Methods:**

Blood samples (300) from the five testing facilities and their screening results for hepatitis B surface antigen (HBsAg), antibodies to hepatitis C virus (HCV) and human immunodeficiency virus (HIV) using RIAs were obtained. All the samples were then analysed for the three viral markers using 3^rd^ generational enzyme linked immunosorbent assay (ELISA) kit as the gold standard.

**Results:**

The mean false positive for HBsAg was 2.2% with Bekwai testing facility having the highest of 4.4%. For HCV, the mean false positive was 2.8% with Agogo and Bekwai testing facilities having the highest of 8.7% respectively. For HIV screening, the mean false positive was 11.1% with Bekwai testing facility having the highest of 28.0%. The mean false negative for the facilities were 3.0% for HBV, 75.0% for HCV and 0.0% for HIV with KATH having the highest of 6.3% for HBV, Bekwai having the highest of 100% for HCV and no facility showing false negative for HIV. Mean sensitivity of the screening procedure for the facilities was 97.0%, 25.0% and 100.0% whilst the mean specificity was 97.8%, 97.2% and 88.9% for HBV, HCV and HIV respectively. Statistical comparison among the testing facilities showed no significant differences among the various testing centres for HBV screening; however, significant differences were obtained for HCV and HIV screening.

**Conclusion:**

This study has shown that there is no standardised screening procedure for blood bank testing facilities in the country. There is therefore an urgent need for an internal and external control body to oversee screening procedures in blood banks across the country.

## Introduction

Transfusion transmitted infections (TTIs) still remain major public health problem encountered by the health delivery systems in many developing countries mainly due to under resourced facilities and lack of requisite staff [1]. Blood transmitted infections involving pathogenic viruses are the most prominent in transfusion medicine [2]. In spite of all the scientific advancements and improvements in technology aimed at improving the safety of blood donation, viruses and bacteria mainly hepatitis B virus (HBV), hepatitis C virus (HCV) and human immunodeficiency virus (HIV) and syphilis still remain the most transmitted infectious pathogenic agents passed on from donor blood to recipient through transfusion [3]. In 2001, the World Health Organisation (WHO) estimated that transfusion of unsafe blood accounted for 8 - 16 million hepatitis B virus infections, 2.3 - 4.7 million hepatitis C infection, and 80,000 - 160,000 human immunodeficiency virus (HIV) infections each year [4]. Even though there are routine tests for these viruses, the greatest risk are donations given in the infectious window period (WP), which is the time between development of infectious viraemia and reactivity by routine serological or nucleic acid technology (NAT) donor screening tests [5]. Blood donors like anyone else, could occasionally carry an infectious agent, sometimes for a long period without having any clinical signs or symptoms [6] thus possessing threat to future recipients through the blood products from such donors. Several screening tests/assays have been developed over the years to overcome this threat; Some of these assay techniques used in screening blood/blood products prior to transfusion include immunochromatographic assay, enzyme-linked immunosorbent assay (ELISA) and nucleic acid test (NAT) or polymerase chain reaction (PCR) assay techniques. The effectiveness of all these tests in interdicting contaminated units of blood/blood products depends in part on the point in time when an infected donor provides the unit relative to the individual's exposure to the virus [7], adequate training of laboratory staff on risks of transfusion-associated viral transmission and also their ability to follow standardised procedures in applying all these testing technologies as well as adhering to good quality control measures so as to prevent testing/procedural errors [5, 8, 9].

Issues concerning blood safety are mostly in two parts: how to identify infectious donor or blood unit and thus prevent its onward transfusion in so doing safeguarding the recipient; and also how to prevent false positives which result in deferment of potential blood donors or discarding of donated blood. The success or otherwise of such an action depends on two main policies. The first strategy is the adaptation of a national transfusion policy for the selection of blood donors which mainly aims at excluding blood donors with higher risk for infections like HIV, HBV, HCV and syphilis. The second is the application of assay technologies with high specificity and sensitivity so as to be able to identify all true positive individuals/blood units and true negative individuals/blood units [10]. Screening of blood donors is a critical issue as the outcome of the test if not properly performed can result in serious consequences for either the blood service or the blood donor. False positive result can lead to a larger number of blood donors being deferred, while a false negative testing may jeopardize blood safety [11]. Improperly trained laboratory personnel may also produce a false screening result. Transfusion of an infected blood to an individual is a crime [12] and to avoid this, strict haemovigilance and quality control systems (both internal and external) need to be put in place in all blood centre facilities. Currently in Ghana, the policy of blood donor selection include or exclude a donor on the basis of a health check questionnaire and a visual physical examination together with a mandatory screening for HIV, HBV, HCV and syphilis [13]. But in Ghana and most sub-Saharan Africa countries, resources for viral marker screening among blood donor are limited, with the exception of immunodeficiency virus screening which is mostly supported by long-term foreign aid [14]. Antibodies to hepatitis C and hepatitis B surface antigen (HBsAg) even though routinely done do not enjoy such attention and supervision hence the type of assay technology and brand of test kit used in a blood facility is the choice solely of the individual facilities. Most facilities currently use lateral flow immunochromatographic rapid tests or enzyme immunoassays (EIA) test kits in the screening of blood donors for viral markers [13, 15]. Currently there is limited information/data on the evaluation of the residual risk of viral transmission in Ghana, but Allain et al (2003) observed that neither the dipstick assay nor latex agglutination assays currently in use for HBsAg screening have sufficient sensitivity in detecting HBV in infected blood, with latex agglutination and dipstick methods presenting a false negative rate of 46% and 29% respectively. The primary objective of this study therefore was to retest samples from five testing facilities who used rapid immunochromatographic assay to screen blood donors with enzyme linked immunosorbent assay (ELISA), which is the method recommended by WHO for all blood banks [16] and compare the results from the various facilities with ELISA results.

## Methods

Blood samples for the study were collected from testing facilities of the following hospitals: Komfo Anokye Teaching Hospital - Kumasi, Bekwai Government Hospital - Bekwai, Agogo Presbyterian Hospital - Agogo, Kwame Nkrumah University of Science and Technology Hospital - Kumasi, all in the Ashanti region and Sunyani Regional Hospital - Sunyani in the Brong Ahafo region. Approval for the study was granted by Committee on Human Research Publication and Ethics, School of Medical Sciences - KNUST. In addition approval was also obtained from the authorities of the various hospitals involved in the study. All the samples were tested for the three viral markers (HBV, HCV and HIV) using rapid immunochromatographic assays at their respective testing facilities. The results of the testing using RIA and the type of test kit used at each of the testing facility were collated on to a case report form. Plasma/serum from each of the samples were separated into well-labelled micro-tubes and then transported in cold boxes to Komfo Anokye Teaching Hospital and stored at -20 °C till ELISA assaying was performed. ELISA assaying was performed using one-step incubation, double antibody sandwich enzyme-linked immunosorbent assay (ELISA) manufactured by Innovation Biotech Co., Ltd Beijing - China. The ELISA assaying of samples were carried out as described by the manufacturer.

## Results

### Reactivity of the viral markers at the various testing facilities upon re-testing with ELISA

As shown in Table 1, 300 samples were analysed during the study, of which 100 came from KATH and 50 each from KNUST, Bekwai, Agogo and Sunyani blood banks respectively. When the samples were retested with ELISA, 6 (2.2%) were false positives and 1 (3.0%) false negative for HBV. Similarly, 8(2.8%) and 9(75.0%) were false positives and false negative respectively for HCV and 33 (11.1%) false positives for HIV. Bekwai testing facility had the highest 2(4.4%) false positives whilst KATH has the highest 1(6.3) false negative for hepatitis B testing. For hepatitis C virus testing, Agogo and Bekwai testing facilities had the highest 4(8.7%) false positives. Bekwai testing facility showed the highest 4(100%) false negatives. For HIV testing, Bekwai testing facility showed the highest 14 (28.0) of false positives with none of the centres showing any false negative testing result.

**Table 1:** Rapid immunochromatographic assays and ELISA for hepatitis B Virus, hepatitis C virus and human immunodeficiency virus stratified by testing facilities

Parameter	Hepatitis B Virus				Hepatitis C Virus				Human Immunodeficiency Virus			
	Elisa Negative	False Positive	Elisa Positive	False Negative	Elisa Negative	False Positive	Elisa Positive	False Negative	Elisa Negative	False Positive	Elisa Positive	False Negative
Total	267	6(2.2)	33	1(3.0)	288	8(2.8)	12	9(75.0)	297	33 (11.1)	3	0(0.0)
Agogo	43	1(2.3)	7	0(0.0)	46	4(8.7)	4	3(75.0)	49	10 (20.4)	1	0(0.0)
Bekwai	45	2(4.4)	5	0(0.0)	46	4(8.7)	4	4(100)	50	14 (28.0)	0	0(0.0)
KATH	84	3(3.6)	16	1(6.3)	96	0(0.0)	4	2(50.0)	99	6 (6.1)	1	0(0.0)
KNUST	49	0(0.0)	1	0(0.0)	50	0(0.0)	0	0(0.0)	50	0 (0.0)	0	0(0.0)
Sunyani	46	0(0.0)	4	0(0.0)	50	0(0.0)	0	0(0.0)	49	3 (6.1)	1	0(0.0)

Data are presented as number or number (percentage). Elisa = Enzyme immunosorbent assay; KATH = Komfo Anokye Teaching Hospital; KNUST = Kwame Nkrumah University of Science and Technology

### Specificity and sensitivity of rapid immunochromatographic assays (RIAs)

The specificity of a testing facility is defined as the proportion of population/subjects without an infection that the testing facility will truly confirm negative for the infection being tested whilst the sensitivity of a testing facility is the defined as the proportion of population/subjects with an infection that the testing facility will truly confirm positive for the infection being tested [17]. The specificity and sensitivity for each of the testing facility were also calculated as shown in Table 2. The mean specificity and sensitivity for the facilities were 97.8% and 97.0% for hepatitis B virus respectively, 97.2% and 25.0% for Hepatitis C virus respectively and 88.9% and 100% for human immunodeficiency virus respectively. Bekwai testing facility had the lowest (95.6%) specificity for HBV; Agogo and Bekwai facilities had the lowest (91.3%) specificity for HCV whilst Bekwai had the lowest (72.0%) for HIV. For sensitivity, KATH had the lowest (93.8%) for HBV; Bekwai had the lowest (0.0%) for HCV with all the centres having 100% sensitivity for HIV.

**Table 2:** Accuracy of rapid immunochromatographic assays against ELISA stratified by testing facility

Parameter	Specificity	Sensitivity	J-Index	PPV	NPV	LR+	LR-	RR
**Hepatitis B virus**								
Total	97.8	97.0	1.0	84.2	99.6	43.2	0.0	8.11 (0.01-16.65)
Agogo	97.7	100	1.0	87.5	100	43.0	0.0	0.00 (0.00-40.44)
Bekwai	95.6	100	1.0	71.4	100	22.5	0.0	0.00 (0.00-48.91)
KATH	96.4	93.8	0.9	83.3	98.8	26.3	0.1	15.0 (0.01-30.31)
KNUST	100	100	1.0	100	100	NA	0.0	0.00 (0.00-83.25)
Sunyani	100	100	1.0	100	100	NA	0.0	0.00 (0.00-54.59)
**Hepatitis C Virus**								
Total	97.2	25.0	0.2	27.3	96.9	9.0	0.8	68.75 (46.15-91.73)
Agogo	91.3	25.0	0.2	20.0	93.3	2.9	0.8	62.50 (28.91-96.59)
Bekwai	91.3	0.0	-0.1	0.0	91.3	0.0	1.1	75.00 (45.41-100)
KATH	100	50.0	0.5	100	98.0	NA	0.5	50.00 (15.00-85.00)
KNUST	100	NA	NA	NA	100	NA	1.0	NA
Sunyani	100	NA	NA	NA	100	NA	1.0	NA
**Human Immuno virus**								
Total	88.9	100	0.9	8.3	100	9.0	0.0	0.00 (0.00-61.75)
Agogo	79.6	100	0.8	9.1	100	4.9	0.0	0.00 (0.00-83.25)
Bekwai	72.0	NA	-0.5	0.0	100	0.0	1.4	NA
KATH	93.9	100	0.9	14.3	100	16.5	0.0	0.00 (0.00-83.25)
KNUST	100	NA	NA	NA	100	NA	1.0	NA
Sunyani	93.9	100	0.9	25.0	100	16.3	0.0	0.00 (0.00-83.25)

Data are presented as number. ELISA = enzyme immunosoebent assay; J-Index = Youden's statistic Index; LR+ = likelihood ratio positive; LR- = likelihood ratio negative; RR = residual risk; KATH = Komfo Anokye Teaching Hospital; KNUST = Kwame Nkrumah University of Science and Technology; NA = could not be calculated.

### The youden index for acceptability of testing methods

The Youden index also known as the J-index [18–19] was used to assess the accuracy or the acceptability of assaying results from each of the testing facilities. The higher the J-index of a facility, the better is the accuracy of the result from that facility as well as the more acceptable the result. J-index has an upper limit of 1.0. From Table 2, the average calculated J-index for HBV assaying at the various facilities was 1.0, with KATH having a value 0.9 which is lower than the average. The calculated average J-index for HCV screening at the various facilities was 0.2 with KATH having a value of 0.5 which is far above the facility average whilst Bekwai had a value of -0.1 which was much lower than the facility average. For HIV testing at the various facilities, the average J-index was 0.9 with Bekwai testing facility having value of negative 0,5.

### Positive Predictive Value (PPV)

PPV defines the proportion of individuals with a positive test result who do actually have the disease/infection [17]. PPV is calculated by using the formula ( TP/ (TP+FP)’>TP/ (TP+FP)) and it gives the proportion of people with positive test result in a population of people with positive result [20]. The higher the PPV of an assay/facility, the better its ability to give a positive test result. KNUST and Sunyani testing facilities had the highest (100%) positive predictive value/rate for HBV testing, whilst Bekwai testing facility had the lowest rate of 71.4% (Table 2). For HCV testing, Bekwai testing facility had the lowest rate of 0.0% whilst KATH had the highest rate of 100% (Table 2). For HIV testing, Sunyani testing facility showed the highest PPV of 25.0% whilst Bekwai testing facility had the lowest rate of 0.0% (Table 2).

### Negative Predictive Value (NPV)

NPV of a test is the proportion of people with a negative test result who do not have the disease/infection being tested [17]. NPV is calculated by using the formula ( TN/(TN+FN)’>TN/(TN+FN)) and its gives the proportion of subjects without a disease who have a negative test result in a population of individuals with a negative test results [20]. Agogo, Bekwai, KNUST and Sunyani testing facilities had the highest (100%) negative predictive value/rate for HBV testing, whilst KATH testing facility had the lowest rate of 98.8% (Table 2). For HCV testing, Bekwai testing facility had the lowest NPV of 91.3% whilst KNUST and Sunyani had the highest rate of 100% (Table 2). For HIV testing, all the testing facilities showed the same level of NPV of 25.0% (Table 2).

### Positive Likelihood Ratio (LR+)

The odds ratio that a positive test result will be observed in an infected population compared to the odds that the same result will be observed among a non-infected population also known as the positive likelihood ratio was calculated for each of the testing facilities [20]. Agogo facility had the highest (43.0) positive likelihood ratio for HBV testing, whilst Bekwai facility had the lowest rate of 22.5 (Table 2). For HCV testing, Bekwai facility had the lowest rate of 0.0 whilst Agogo had the highest rate of 2.9 (Table 2). For HIV testing, KATH facility showed the highest positive Likelihood ratio of 16.5 whilst Bekwai facility had the lowest rate of 0.0 (Table 2).

### Negative Likelihood Ratio (LR-)

The odds ratio that a negative test result will be observed in an infected population compared to the odds that the same result will be observed among a non-infected population also known as the negative likelihood ratio (LR-) [20], was determined for each of the testing facilities. KATH testing facility had the highest (0.1) negative likelihood ratio for HBV testing, whilst Agogo, Bekwai, KUNST and Sunyani facilities had the lowest rate of 0.0 (Table 2). For HCV testing, KATH facility had the lowest rate of 0.5 whilst Bekwai had the highest rate of 1.1 (Table 2). For HIV testing, Bekwai facility showed the highest negative Likelihood ratio of 1.4 whilst Agogo, KATH and Sunyani facility had the lowest rate of 0.0 (Table 2).

### Residual risk of transmission of an infection through transfusion

The residual risk of transmission of an infection through transfusion is the probability that a recipient of blood transfusion will get an infection from the blood transfusion the even though the blood had been tested for that infection. The modified Wald analysis [21] was used to estimate the residual risk of transmission of the various viruses (HBV, HCV and HIV) through blood transfusion at each of the testing facilities (Table 2). For HBV testing the mean residual risk of transmission was 8.11% (0.01% - 16.65% at 95% CI) with KATH testing facility generated the highest residual risk of transmission, 15.50% (0.01% - 30.31% at 95% CI). For HCV, the mean residual risk of transmission was 68.75% (46.15% - 91.73% at 95% CI) with Bekwai testing facility generated the highest value of 75.00% (45.41% - 100.00% at 95% CI) residual risk of transmission of HCV which is greater than the mean value. The residual risk of transmission of HIV was 0.00% (0.00% - 83.25% at 95% CI). For HIV testing, there was no mean residual risk of transmission.

### Comparison of testing facility (RIAs) and ELISA using Bland-Altman estimation

The Bland-Altman analysis [22] was used to compare the testing facilities based on their testing bias or degree of estimation in using RIAs rather than ELISA in testing for the presence of the three viral markers. As shown in Table 3, Bekwai testing facility showed the highest underestimation of -0.04 for hepatitis B virus testing, Agogo testing facility showed the highest underestimation of -0.02 for hepatitis C virus whilst Bekwai testing facility showed the highest underestimation of -0.27 for human immunodeficiency virus.

**Table 3:** Bland and Altman estimated bias of rapid immunochromatographic assays against ELISA stratified by facility

Parameter	Bias (HBV)	Bias (HCV)	Bias (HIV)
Total	-0.03 (-0.39 to 0.34)	0.01 (-0.57 to 0.58)	-0.16 (-0.87 to 0.56)
Agogo	-0.02 (-0.30 to 0.26)	-0.02 (-0.76 to 0.72)	-0.24 (-1.08 to 0.61)
Bekwai	-0.04 (-0.43 to 0.35)	0.00 (-0.79 to 0.79)	-0.27 (-1.17 to 0.61)
KATH	-0.02 (-0.41 to 0.37)	0.02 (-0.26 to 0.30)	-0.06 (-0.53 to 0.41)
KNUST	0.00	0.00	-0.02 (-0.30 to 0.26)
Sunyani	0.00	0.00	-0.06 (-0.53 to 0.41)

Data is presented as figures at 95% confidence interval in parenthesis. ELISA- Enzyme linked immunosorbent assay; HBV- Hepatitis B virus; HCV- Hepatitis C virus; HIV- Human immunodeficiency virus; KATH- Komfo Anokye Teaching Hospital; KNUST- Kwame Nkrumah University of Science and Technology

### Performance comparison of testing facilities

The numbers of false positive and false negative results reported by each facility for the various RIAs were used to do a performance comparison among them. Table 4 and Table 5 showed that there was no significant difference between all the facilities with respect to HBV testing (p > 0.05), however there was significant difference (p = 0.0066) between Agogo and KATH testing facilities respect to HCV testing with KATH facility performing better. There was also significant difference between Agogo and KNUST (p = 0.0125) and Agogo and Sunyani (p = 0.0125) for HCV testing with Agogo facility performing worse in both cases. For HIV testing, there were significant differences between Agogo and KATH (p = 0.0124), as well as Agogo and KNUST (p = 0.0012) testing facilities with Agogo performing worse. There was a significant difference between Bekwai and KATH (p = 0.0005), KNUST (p <0.0001) and Sunyani (p = 0.0064) facilities with Bekwai facility performing poorly in all cases.

**Table 4:** Comparison of facility performance for viral serological markers. upper right handed side represents HBV testing and the lower left represent HCV

Facility	Agogo	Bekwai	KATH	KNUST	Sunyani
Agogo		1.0000	0.6652	1.0000	1.0000
Bekwai	0.5246		1.0000	0.4949	0.4949
KATH	0.0066	0.0957		0.3017	0.3017
KNUST	0.0125	0.1175	0.5526		ND
Sunyani	0.0125	0.1175	0.5526	ND	

Data is presented as p-values. p is significant at < 0.05. ND = Note done. KATH = Komfo Anokye Teaching Hospital, KNUST = Kwame Nkrumah University of Science and Technology

**Table 5:** Comparison of facility performance for HIV testing

Facility	Agogo	Bekwai	KATH	KNUST	Sunyani
Agogo					
Bekwai	0.4829				
KATH	0.0124	0.0005			
KNUST	0.0012	<0.0001	0.1790		
Sunyani	0.0713	0.0064	1.0000	0.2424	

Data is presented as p-values. p is significant at < 0.05. KATH = Komfo Anokye Teaching Hospital, KNUST = Kwame Nkrumah University of Science and Technology

## Discussion

Safe transfusion practice has been a matter of concern for the health care providers since the commencement of blood transfusion practices and the subsequent discovery of blood transmissible infectious agents [23]. To ensure safe transfusion practices across the world, the World Health Organisation (WHO) declared 2012 as the year for attaining 100% testing for infectious viral markers in blood products [24]. The status of this target in most developing countries especially Sub-Saharan Africa is however unknown presently as the region is burdened with a lot of social and scientific factors that works against safe transfusion practices [24, 25]. From the survey of this study, it was observed that all the testing facilities screened blood donors for hepatitis B virus, hepatitis C virus and human immunodeficiency virus using rapid test strip rather than ELISA recommended by World Health Organisation [26]. The result from our study showed a discrepancy between the results obtained at the various testing facilities using rapid immunological assays compared to what was obtained when ELISA was used to retest the samples. The various facilities analysed in the study showed a mean false positivity of 2.2%, 2.8% and 11.1% for HBV, HCV and HIV testing respectively when compared with ELISA (Table 1). These rates are in the range of what was obtained in other studies [25, 27, 28]. The mean false negative was 3.0%, 75.0% and 0.0% for HBV, HCV and HIV respectively. The high percentage of false negative for HCV compared to false positive may be attributed to deficient detection of the genotypes and/or subtypes with the type of rapid assay used which supports the reason why all rapid tests must be evaluated to meet the local standard before being used as a screening assay. From Table 2, the total specificity (97.8%) and sensitivity (97.0%) were higher compared to the total PPV of 84.3% but lower than the NPV of 99.6%. This gives an indication that the testing facilities were producing more false positives than false negative in their screening. In blood bank settings, false positive results most often than not has negative consequences on blood donors as this will drive way a lot of potential donors thereby decreasing the blood stock of a facility. However, several studies have suggested that false positives especially in the case of HCV testing can never be completely ruled out especially in Africa where Africans have be shown to produce antibodies that react non-specifically with most rapid tests and lower generations of ELISA a phenomenon that has necessitated the development of newer generations of ELISA [27, 29]. The residual risk of transmission of HBV, HCV and HIV as a result of the use of RIA was estimated using the Modified Wald method [21]. The mean estimated risk of 8.11% is similar to what Owiredu et al. (2012) reported (8.47%) as the risk of transmission of transfusion borne HBV to recipients by the use of immunochromatographic assays in ten (10) regional hospitals of Ghana. The high residual risk may be attributed to either the facilities are not carrying out the testing procedure competently or the rapid immunochromatographic assays are performing below standards and therefore are not able to detect some of the viral markers [10]. This indicates that there is more work to be done if we are to totally prevent transfusion transmissible infections.

Diagnostic tests are considered to have poor diagnostic accuracy if their J-index is equal zero and below [20]. Bekwai facility had the lowest diagnostic accuracydue to a J-index of -0.1 for HCV testing and -0.5 for HIV testing (Table 2). Bekwai testing facility was the worst performer in predicting both positive (an LR+ of 22.5) and negative (LR- of 0.0) for HBV test results among individuals with or without an infection respectively. The performance of an assay is also greatly affected by factors such as the technical expertise of person performing test, storage of the kits and technique used during the assaying process. This may account for the varied degrees of biases obtained even though the same type of assay brand (First Response^®^) was used in the testing facilities (Agogo, Bekwai, and Sunyani) in screening for HIV (Table 3). For HBV and HIV testing, negative biases were obtained similar to what was obtained by Owiredu et al. (2012) for the use of rapid test verses ELISA in the screening of blood donors for HBsAg among testing facilities in some regional hospitals of Ghana, an indication that the facilities are under estimating the prevalence of these markers among the donor population, which may give rise to the transmission of these infections to blood recipients. For HCV testing, a mean positive bias was obtained, an indication that the facilities were over reporting the prevalence of HCV among the donor population resulting in more blood donors being deferred. This study also revealed that a facility may use a brand of rapid immunochromatograhic assay depending on its affordability or availability in the open market. The call made by Allain et al., (2003) for a centralised control on standardised guidelines on the acquisition of test kits and a national policy geared toward ensuring the quality of pre-screening of viral markers before transfusion, should not be underestimated if we are to completely prevent transfusion transmittable infections. Comparison among the testing facilities was done based on the number of false positives and false negatives reported by each facility. Generally there was no statistical significant difference (p > 0.05) among the five facilities relative to HBV testing however some facilities did better than others with respective to HCV and HIV testing. The comparison showed significant difference (p < 0.05) in the performance of Agogo testing facility to that of KATH, Sunyani and KNUST facilities with Agogo performing worst in all cases. For HIV testing, there was also significant statistical difference in performance with Agogo doing worst than KATH and KNUST and Bekwai testing facility also doing poorer than KNUST, KATH and Sunyani. These comparisons gave an indication that the probability of a recipient of transfusion acquiring an infection through blood or a blood being rejected for blood donation is higher in some facilities than others and there is still more work to be done if all our testing facilities are to be at par. Even though RIAs are useful screening methods in blood bank testing facilities in low-resource settings however, the method should be carefully validated locally before being used for screening as their performance may varies by location.

## Conclusion

The evaluation of the performance of five hospital based testing facilities in the use of rapid immunochromatograhic assays to screen blood donors for HBV, HCV and HIV in Ghana showed that the strategy being used by the facilities now is inadequate to prevent transmission of HBV, HCV and HIV through blood transfusion in Ghana due to high residual risk of transmission of these viruses through blood transfusion. There is thus an urgent need for an effective and efficient control system to be put in place for screening of blood donors at these and other facilities throughout the country.
